# An Information-Theoretic Method for Identifying Effective Treatments and Policies at the Beginning of a Pandemic

**DOI:** 10.3390/e26121021

**Published:** 2024-11-26

**Authors:** Amos Golan, Tinatin Mumladze, Jeffery M. Perloff, Danielle Wilson

**Affiliations:** 1 Department of Economics, American University, Washington, DC 20016, USA; tm3538a@american.edu (T.M.); dw8768a@american.edu (D.W.); 2 Santa Fe Institute, Santa Fe, NM 87501, USA; 3 Department of Agriculture & Resource Economics, University of California Berkley, Berkeley, CA 94720, USA; jperloff@berkeley.edu

**Keywords:** modeling with insufficient information, information-theoretic inference, prediction, COVID-19, mortality, pandemic

## Abstract

Identifying effective treatments and policies early in a pandemic is challenging because only limited and noisy data are available and biological processes are unknown or uncertain. Consequently, classical statistical procedures may not work or require strong structural assumptions. We present an information-theoretic approach that can overcome these problems and identify effective treatments and policies. The efficacy of this approach is illustrated using a study conducted at the beginning of the COVID-19 pandemic. We applied this approach with and without prior information to the limited international data available in the second month (24 April 2020) of the COVID-19 pandemic. To check if our results were plausible, we conducted a second statistical analysis using an international sample with millions of observations available at the end of the pandemic’s pre-vaccination period (mid-December 2020). Even with limited data, the information-theoretic estimates from the original study performed well in identifying influential factors and helped explain why death rates varied across nations. Later experiments and statistical analyses based on more recent, richer data confirm that these factors contribute to survival. Overall, the proposed information-theoretic statistical technique is a robust method that can overcome the challenges of under-identified estimation problems in the early stages of medical emergencies. It can easily incorporate prior information from theory, logic, or previously observed emergencies.

## 1. Introduction

When the next pandemic strikes, how can we choose treatments and policies to reduce deaths before a new vaccine is available? Eventually, we will have a plethora of data and an understanding of the relevant biology, so we can use standard statistical techniques to determine what we should have done using 20−20 hindsight. Unfortunately, at the start of a pandemic, we have few observations and a limited understanding of a disease, so traditional statistical methods may be infeasible or require strong, possibly inaccurate assumptions. Our objective is to propose a new information-theoretic inferential model that can work well and is helpful at the onset of pandemics using only a small number of observations and does not require imposing heroic conjectures.

We demonstrate the efficacy of this approach in an analysis conducted in May 2020 using data from the first few months of the COVID-19 pandemic, when only 485 individual observations from 20 countries were available [1]. Using those data in conjunction with other country-specific data, the study identified two factors—an existing vaccine and pollution levels—associated with lower COVID-19 death rates. Later published experiments and a statistical analysis based on a richer dataset of millions of observations available much later confirm the original study’s conclusions and that these factors contribute to survival. Both these potential policies could have been implemented in the short run. 

This paper’s purpose is to show that an information-theoretic approach can be useful early in a crisis before extensive data are available. However, because crises differ, one might study policies other than those examined here in future crises.

## 2. Methods

Statistical inference with uncertainty and little information results in multiple possible solutions, each consistent with the observed information because the problem is underdetermined. A simple example is the common problem of inferring a *T*-dimensional probability distribution function from a small number, *M*, of known quantities (say, moments) with more unknown probabilities than observed moments, so *M* < *T*. The principle of Maximum Entropy [2,3,4] uses the available information (the *M* moments and normalization) as constraints in an optimization problem to select a solution using Shannon entropy [5] as the decision function. The maximum entropy solution is the least-biased approach. It is not biased by structural modeling assumptions. It is the flattest, and therefore least informative, probability distribution (likelihood) compatible with the information captured in the constraints [6,7,8]. Stated differently, out of all solutions consistent with the information we have, this method selects the most uniform—uninformed—one.

But, the classical Maximum Entropy (ME) formalism may not work with model ambiguity and insufficient, noisy, and complex information, such as the information at the onset of a new pandemic. However, an information-theoretic approach, which generalizes the ME, accommodates these challenges (see Appendix A and the Supplementary Materials). In the absence of these complications, the solution of this information-theoretic approach converges with that of the ME.

This approach incorporates each piece of information as a flexible constraint with additive mean-zero uncertainty. It maximizes the Shannon entropy decision function defined over the probabilities of interest (here, the survival rate), accounting for the uncertainties in the constraints. It can be applied even with few observations and little or no knowledge of the underlying biological model. See Appendix A for the detailed derivation.

The binary choice information-theoretic approach we used dominates the classical maximum likelihood for finite samples (lower variance and better prediction) and allows us to use informative priors, significantly improving the inference [9]. The informative priors may reflect fundamental principles, logical reasoning, or empirical observations. Empirical priors must be independent of the data used for the analysis but capture the universal characteristics and features of the population of interest [10]. In our application, our informative priors are observed death frequencies by age and sex for individuals previously infected with SARS because different coronaviruses with similar characteristics cause SARS and COVID-19 [11]. Our application demonstrates that this choice of priors improves the model’s in- and out-of-sample predictions.

At the beginning of the COVID-19 pandemic, we applied this approach to identify existing treatments and policies that could reduce death rates. The patients’ data came from the Open COVID-19 Data Curation Group [12], which had only a small amount of publicly available patient-level data as of 24 April 2020. Although the disease had infected millions, the dataset contained only 485 infected individuals from 20 countries with the age, sex, and survival information—the minimal information necessary for our analysis. We supplemented this dataset with country-specific information about BCG−tuberculosis and polio vaccination policies, public health policies, pollution levels, education, and economic characteristics. (Because the polio vaccination, education, and economic variables were not statistically significant, we do not report them in the following results.) Polio and BCG vaccinations were used because they are well-studied and known to positively affect the immune system (especially the BCG) beyond their original purpose (e.g., [13]). Of course, more health and other individual-specific information would have provided more efficient estimates, but they were not available.

The binary dependent variable is one if an infected individual died or zero if the individual survived (“discharged from the hospital” or “recovered”). We did not include individuals whose outcome was unclear (e.g., they were still sick) at the time of our analysis.

We use two binary BCG policy variables [14]. The first equals one if a country never had a universal vaccination program (e.g., the United States) and zero otherwise. The second equals one if a country’s former BCG policy ended before the pandemic (e.g., Australia). The base case is a current BCG policy (e.g., the Philippines).

Our environmental variable is the air pollution death rate. The World Bank estimates the annual deaths attributable to household and ambient air pollution [15].

We used three health variables for each country: the World Health Organization’s estimate of the domestic private health expenditure per capita (in international dollars at the purchasing power parity) and the measles and hepatitis B immunization rates [15]. We did not expect those vaccinations to affect COVID-19 outcomes directly but viewed them as proxies for health policies in general. Table S1 in the Supplementary Materials summarizes the dataset used and its resources.

We estimated (in early 2020) an information-theoretic [1] binomial model (Appendix A) to infer the survival probability of an infected individual, conditional on age, sex, and country-specific factors with and without priors.

To validate the performance of our model in retrospect, we (i) contrasted the results of the original small sample with another analysis using millions of observations collected later in the pandemic and (ii) validated our results with recent published experiments and studies.

## 3. Results

Using the small dataset available early in the pandemic, we estimated two types of models. One did not use informative priors. (It assumes uninformative priors with a uniform distribution—everything is a priori equally probable.) The second uses informative priors based on SARS. Both models fit the data well, but the model with informative priors produces more accurate predictions. Table 1 shows our estimated coefficients and confidence intervals (CI) for the model with informative priors. (The coefficients and confidence intervals for the other model are qualitatively similar.) All estimated coefficients except for females and measles are statistically significantly different from zero at the 0.05 level. The pseudo-R^2^ is 0.54.

The table’s last column shows the marginal effects of each variable: the change in the probability of dying due to a change in this explanatory variable holding other variables constant at their means. For example, compared to a country with a current BCG program, the probability of dying would be 45% higher (with a 95% confidence interval of [36.0%, 54.6%]), holding other variables constant.

To see how well the model predicts, we used a *k*-fold cross-validation method. First, we use a uniform distribution to randomly assign a number to each sample observation and place the observations in ascending order according to their randomly assigned number. Second, we split the sample into *k* subsets (folds) of approximately the same size. Third, we estimate our information-theoretical model using all observations but the first subset. Fourth, we use these estimated coefficients to predict whether an individual in the first fold died if the probability exceeds 0.5 (the standard threshold). Fifth, we repeat the process, changing the test subset for the remaining *k* − 1 folds.

Table 2 reports the average for 10 replications of the k-fold analysis for k = 10 (the results were qualitatively similar for different choices of k). The values reported reflect averaging over the actual and predicted deaths and then averaging over the entire sample (all the folds). The model’s correct classification score for the survival variable is 83.3%. The sensitivity score (actual ones—die—are correctly predicted) is 98.4%. The specificity score (actual zeros—survive—are correctly predicted) is 72.3%. These results for the model without informative priors were similar but did not predict as well.

We focus on the effects of pollution and a BCG vaccination on COVID-19 patients’ survival probabilities. A ten percentage point increase in air pollution raised the probability of death from the COVID-19 virus by 2.6 percentage points evaluated at the other explanatory variables’ means other than age.

**Figure 1:**
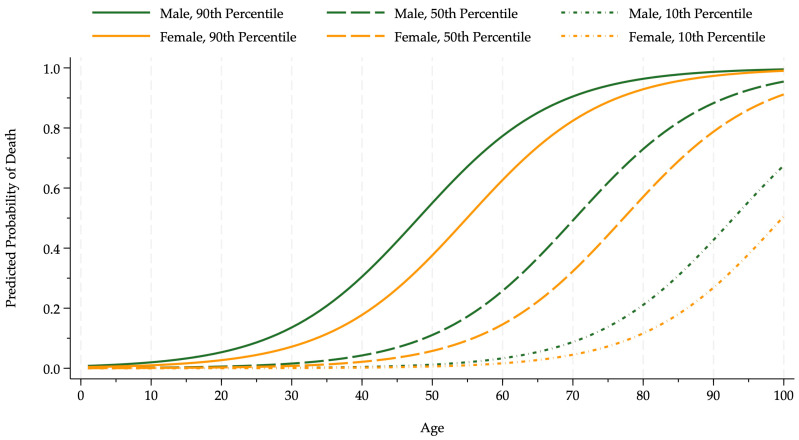
Pollution–death probability by sex and age. The figure shows the probability that an infected COVID-19 patient died for age and sex groups (females, orange; males, green) conditional on pollution at low (10th percentile, dotted line), median (50th percentile, dashed line), and high (90th percentile, solid line) levels.

Figure 1 shows how pollution affects the death probabilities, controlling for all other factors and vaccines. The death probability curves rise sharply with age. The curves evaluated at the ninetieth pollution percentile lie substantially above those at the median and the tenth. The women’s curves lie below the men’s.

**Figure 2:**
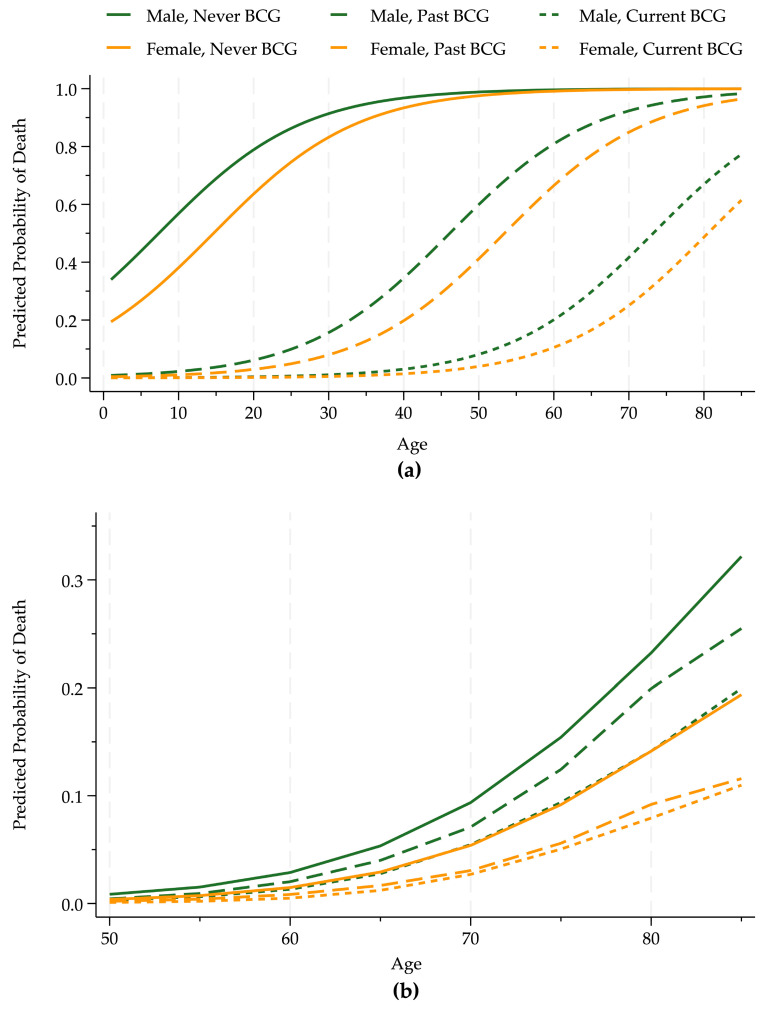
BCG–death rate by age and sex. Panel (**a**) shows the estimated probability that an infected COVID-19 patient died for age and sex groups (males, dark green, and females, light orange) conditional on BCG vaccination policies using data through 24 April 2020. Panel (**b**) illustrates the cumulative frequency of the death of infected individuals over 50 years old using data from the beginning of the pandemic through mid-December 2020, the pre-vaccine period (approximately 12 million patients), in countries with a low pollution level.

Panel a of Figure 2 shows that current or past BCG vaccine policies substantially increased survival probabilities. At the margin for the middle of the age distribution, men and women from countries that never had a universal BCG vaccination policy were about 50 percentage points more likely to die from COVID-19 than individuals from countries with a current universal policy and about 30 percentage points more likely than those with a previous vaccination policy. The death probability rises substantially with age, and the curves for women lie below those for men.

We used a much larger publicly available dataset [16] from a longer period to determine whether these results based on limited and imperfect information available at the beginning of the pandemic are plausible and qualitatively accurate. Panel b of Figure 2 shows the cumulative frequencies from the pandemic’s beginning through mid-December 2020, the end of the pre-vaccination period for 12,654,066 individuals in eleven countries (Australia, Austria, Canada, Denmark, France, Italy, Japan, Norway, Portugal, Spain, and the United States) with low (10%) pollution levels. This figure includes only low-pollution countries to illustrate the effects of BCG policies, controlling for pollution.

The death rates are much lower in Panel b than in Panel a because these data are primarily from later in the pandemic when medical treatments were much improved. Also, this large sample includes a greater portion of healthy people, who were less likely to die. Nonetheless, the qualitative results are the same as our small sample estimates from early in the pandemic. Our initial results are supported by the much larger data available later in the pandemic: the BCG policies reduced death rates.

For example, we estimated that a 55-year-old man’s death probability was 7.6 times greater in a country that never had a BCG policy relative to one with a current policy in the initial analysis. This ratio for the cumulative frequencies is 7.4. The corresponding ratio for a country with a past BCG policy to a current one was 5.5 using predicted probabilities and 4.5 using frequencies.

## 4. Discussion

This study demonstrates that an information-theoretic model can identify factors that increase patients’ survival using small datasets available early in a pandemic. Our early, small-dataset study identified two factors that reduce death rates. Our subsequent study using a larger (by a factor of 24,000) dataset was consistent with these results.

Moreover, more recent, well-designed experimental evidence also supports these results. Two recent studies [17,18] confirm our earlier finding about the negative impact of pollution on COVID-19 patients. A randomized, double-blinded, placebo-controlled trial to test the efficacy of the BCG vaccine against COVID-19 found that BCG is safe and is approximately 92% efficacious relative to a placebo group [19]. See [13,20,21].

Both controlling pollution and giving BCG vaccines would have been feasible short-run approaches to reducing COVID-19 death rates before vaccines were available. Pollution can be quickly reduced, as several countries demonstrated during the COVID-19 shutdowns. The Chinese quickly reduced PM10 air pollution by 10% in and around Beijing for their 2008 Olympics [22]. Doing so reduced the overall mortality by 8% and had greater effects on the older population.

The BCG vaccine was widely available, and more could be produced quickly. This vaccine becomes effective within approximately four weeks [23,24].

Pandemics and other health crises are different in their intensities and impacts. Each demands its own set of preventative policies. To select short-term policies to minimize damages, a careful data analysis is needed. But very early in a crisis, the available information is limited, imperfect, and noisy. With such data, classical inferential approaches are often not useful. Our information-theoretic approach can always be used for such an analysis. We showed that among the potential policies available and applicable at the beginning of the COVID-19 pandemic, pollution reduction and BCG vaccination were effective. Other crises will require other policies.

## 5. Conclusions

We proposed a new information-theoretic approach that can identify factors affecting patients’ survival probabilities in the face of great uncertainty stemming from limited information about a complex system and few collinear observations at the beginning of a pandemic. This method can allow policymakers to respond before more reliable experimental studies and data are available early in pandemics and before a new vaccine is available. As such, it can potentially save lives.

By comparing our results to later information, we demonstrated that this approach worked well at the beginning of the COVID-19 pandemic. More recent, well-designed experiments support the initial finding. Other statistics, like our *k*-fold analysis, support our initial study.

The same information-theoretic approach can be used in other scenarios where the data are limited and imperfect, and we are uncertain about the underlying physiological and biological processes, such as for emerging diseases.

## Figures and Tables

**Table 1:** Estimated coefficients and marginal effects for the model with priors.

	Coefficients	Marginal Effects
Female	−0.4545	−0.0357
	(−1.163, 0.254)	(−0.091, 0.02)
	z = −1.26	z = −1.26
Age	0.0524	0.0048
	(0.029, 0.075)	(0.002, 0.008)
	z = 4.48	z = 2.89
BCG never	5.9907	0.4528
	(3.85, 8.131)	(0.360, 0.546)
	z = 5.49	z = 9.58
BCG past	2.6471	0.1721
	(0.557, 4.737)	(0.06, 0.284)
	z = 2.48	z = 3.01
Health expenditure	−0.0019	−0.0002
	(−0.003, −0.001)	(−0.0033, −0.0004)
	z = −2.60	z = −1.89
Air pollution	0.0287	0.0026
	(0.014, 0.043)	(0.001, 0.005)
	z = 3.86	z = 2.69
Measles	−0.0925	−0.0085
	(−0.189, 0.004)	(−0.019, 0.002)
	z = −1.88	z = −1.57
Hepatitis B	0.1666	0.0154
	(0.074, 0.259)	(0.003, 0.028)
	z = 3.53	z = 2.37
Constant	−10.9248	
	(−16.761, −5.089)	
	z = −3.67	
Observations	485	
Degrees of freedom	9	
Pseudo R^2^	0.5417	

Note: 95% confidence interval in parentheses.

**Table 2:** Results from averaging ten 10-fold cross-validations.

Evaluation Score	With Informative Priors
*Prediction Success*	
Correct classification rate	82.6%
Sensitivity (actual 1 s are correctly predicted)	98.7%
Specificity (actual 0 s are correctly predicted)	72.3%
Positive predictive value (predicted 1 s that were actual 1 s)	69.3%
Negative predictive value (predicted 0 s that were actual 0 s)	98.9%
*Prediction Failure*	
False positive rate for true survival (actual 0 s predicted as 1 s)	27.7%
False negative rate for true death (actual 1 s predicted as 0)	1.3%
False positive rate for classified death (predicted 1s that are actual 0 s)	30.7%
False negative rate for classified survival (predicted 0 s that are actual 1s)	1.1%

## Data Availability

The original data presented in the study were produced by the Open COVID Data Group [12], were accessed on 24 April 2020, and are available at https://github.com/beoutbreakprepared/nCoV2019. The cleaned data used in this study are available in the corresponding Supplementary Materials.

## Supplementary Materials

The following supporting information can be downloaded at https://www.mdpi.com/article/10.3390/e26121021/s1, Table S1: Data definitions and sources; S1 File: Data dictionary for cleaned data from the Open COVID Data Group [12]; S2 File: Cleaned, full dataset in both Excel and Stata dta form; S3 File: Cleaned usable subsample (485 observations) from the Open Covid Data Group [12]; S4 File: The main Stata do file for the original model [1]; S5 File: The necessary Stata ado file needed to run the original model. (The ado file, published by STATA, was written by Paul Corral, Senior Economist at the World Bank); A few additional reported experiments. Refs [25,26,27] are cited in Supplementary Materials.

## Appendix A

### Appendix A.1. The Information-Theoretic Inferential Model

#### Appendix A.1.1. Definitions and Problem Specification

Consider an experiment consisting of T categorical trials. Each trial produces a single category as an outcome: one of the J unordered categories j=0, 1,…, J−1. Each outcome is coded as a binary list, with a value of one in the position of the realized category and zero in the other positions. The entire experiment can be represented as a matrix $\left\{y_{ij}\right\}\;\left(i=1,\;2,\ldots ,\;T;j=0,\;\ldots ,\;J-1\right)$, where each row has a single non-zero entry. For each trial i, exactly one of the J categories is observed, so $y_{ij}$ equals one if and only if alternative j is observed; it is zero otherwise. That is, $y_{ij}$ is a T×J matrix; a row for each observation i and a column for each unordered category j. In our case, each “trial” is an individual who tested positive for COVID-19. Let the probability of alternative j, on trial i, be $p_{ij}=Prob\left(y_{ij}=1\right)$. To model these probabilities, we assume that the $p_{ij}s$ are related to a set of covariates X (the individuals’ characteristics and other environmental information). That relationship is captured in the nonlinear model:

(A1) $$p_{ij}\equiv Prob\left(y_{ij}=1\;|x_{i};\beta_{j}\right)=F\left(x_{i}^{\tau }\beta_{j}\right)=F\left(\sum_{k=1}^{K}x_{ik}\beta_{kj}\right)\mathrm{for}\mathrm{all}i\mathrm{and}j$$

where $\beta_{kj}$ are unknown parameters or, similarly, $\beta_{j}$ is a (K×1) vector, $x_{i}$ is a K×1 vector of covariates, τ stands for “transpose”, “|” stands for “conditional on”, and F(·) is a function linking the probabilities $p_{ij}$ with the covariates $\underset{k}{\sum }x_{ik}\beta_{kj}$ such that $F\left(\overset{K}{\underset{k=1}{\sum }}x_{ik}\beta_{kj}\right)=p_{ij}>0$ and $\underset{j}{\sum }F\left(\underset{k}{\sum }x_{ik}\beta_{kj}\right)=\underset{j}{\sum }p_{ij}=1$ for i=1, 2, …, T. The function F(·) is nonlinear.

The core interest here lies in inferring the probabilities $p_{ij}$ based on the observed information $y_{ij}$ and the covariates X. The ML estimator for inferring the parameters of that problem is available in most statistical and econometric textbooks and software. However, as we stated earlier, the traditional approaches, like the maximum likelihood probit or logit, require strong distributional assumptions, which the informational-theoretic approach avoids. Moreover, probit and logit are known to predict poorly in the tails of the distributions and for highly collinear data.

#### Appendix A.1.2. The Model—A Summary

We want to avoid distributional assumptions. We also want to incorporate prior information. Our data are ill-behaved: some control variables are highly correlated, though the overall condition number, capturing the overall level of multicollinearity in the data—the right-hand side variables—is not exceptionally high at 55.4. More importantly, we have relatively few observations. Thus, we need an approach that can accommodate these challenges. The information-theoretic approach we use here allows us to accomplish our objectives. It is based on the work of Golan, Judge, and Perloff [9] and the more recent derivations of Golan [10], which builds on the maximum entropy formalism of Jaynes [2] and the work of Shannon [5] on information (communication) theory and Kullback and Leibler [28]. Under that approach, all information is specified as constraints within a constrained optimization process. For a complete description, derivations, historical perspective, and new results, see Golan [10]. The decision (or objective) function is Shannon’s entropy and the Kullback–Leibler entropy divergence measure.

Given our objectives and the fact that there are always more unknown quantities than known quantities, our problem is underdetermined. To solve it, we must first specify the information we have as constraints. That information consists of the following: the observed outcomes (or choices or actions), $y_{ij}$, and the covariates X. We need to connect these observable quantities with the unobserved entities of interest—the $p_{ij}s$. One way of doing this is via the equation

(A2) $$y_{ij}=F\left(x_{i}^{\tau }\beta_{j}\right)+\epsilon_{ij}=F\left(\sum_{k=1}^{K}x_{ik}\beta_{kj}\right)=p_{ij}+\epsilon_{ij},$$

where $\epsilon_{ij}$ is mean zero noise. We want to infer the individual probabilities while allowing for all possible uncertainties.

Given our objective and that (*i*) our information is uncertain or most of the information is concentrated in only some of the categories and (*ii*) the covariates are ill-behaved, the classical maximum entropy and ML methods may not work. No feasible solution exists in the sense that the values of the inferred parameters go to positive or negative infinity. Further, we have no basis for choosing the function *F*, the likelihood.

Though there is no substitute for better information and more data, we need to do our best with the limited information about the virus and the incomplete and noisy data. As a first step, we need to incorporate the covariates in our constraints. One approach is to use the cross-moments. That is,

(A3) $$\sum_{i}x_{ik}y_{ij}=\sum_{i}x_{ik}p_{ij}+\sum_{i}x_{ik}\epsilon_{ij};\;j=0,\ldots ,\;J-1;k=1,\ldots ,\;K$$

where the error terms $\epsilon_{ij}s$ are naturally bounded in the interval [−1, 1] since each $y_{ij}$ is zero or one.

Following [9] and [10], the $\epsilon_{ij}s$ can be reformulated as an expected value of a random variable with a symmetric-about-zero support space of dimension S≥2 and weights W, such that $\epsilon_{ij}=\underset{s}{\sum }w_{ijs}v_{s}$ and $\underset{s}{\sum }w_{ijs}=1$, where *V* is viewed as a discrete random variable with S possible realizations for each i and j, each one with a probability $w_{ijs}$. The a priori expected value of that random variable for each i and j is zero.

Let W be a matrix of the elements $w_{ijs}s$. Then, the constrained information-theoretic model is specified as

(A4) $$\begin{array}{l} \underset{\left\{P,\;W\right\}}{Minimize}\;D(P,W\;||\;P^{0},\;W^{0})=D\left(P\;||\;P^{0}\right)+D\left(W\;||\;W^{0}\right)\\ subject\;to\\ \sum_{i}x_{ik}y_{ij}=\sum_{i}x_{ik}p_{ij}+\sum_{i}x_{ik}\epsilon_{ij}=\sum_{i}x_{ik}p_{ij}+\sum_{i,s}x_{ik}v_{s}w_{ijs};j=0,\;\ldots ,\;J-1;k=1,\ldots K\;\\ \sum_{j}p_{ij}=1;\;j=0,\;\ldots ,\;J-1\sum_{s}w_{ijs}=1;i=1,\;\ldots ,\;T;j=0,\ldots ,\;J-1 \end{array}$$

where $D\left(P\;||\;P^{0}\right)$ and $D\left(W\;||\;W^{0}\right)$ are the Kullback–Leibler information-divergence measure between P and $P^{0}$ or *W* and $W^{0}$ respectively, and $P^{0}$ and $W^{0}$ are prior probabilities. Our analysis uses the SARS frequencies by age and sex as the informed priors $P^{0}$. (Our model without informative priors assumes the *P*^0^ are distributed uniformly). The priors for the uncertainty elements, $W^{0}$, are taken to be uniform always. Note that due to the additivity property of the log function in the entropy function, the left-hand side of the decision function can be separated into the two components of the right-hand side: *P* and *W*. In other words, the *P*s and the *W*s are independent.

Forming the Lagrangian and minimizing yields the following solution:

(A5) $$p_{ij}^{*}=\frac{p_{ij}^{0}\exp\left(\sum_{k}\lambda_{kj}^{*}x_{ik}\right)}{\sum_{j}p_{ij}^{0}\exp\left(\sum_{k}\lambda_{kj}^{*}x_{ik}\right)}\;\equiv \;\frac{p_{ij}^{0}\exp\left(\sum_{k}\lambda_{kj}^{*}x_{ik}\right)}{\Omega_{i}\left(\lambda^{*}\right)}$$

(A6) $$w_{ijs}^{*}=\frac{w_{ijs}^{0}exp\left(v_{s}\sum_{k}\lambda_{kj}^{*}x_{ik}\right)}{\sum_{s}w_{ijs}^{0}exp\left(v_{s}\sum_{k}\lambda_{kj}^{*}x_{ik}\right)}\;\equiv \frac{w_{ijs}^{0}exp\left(v_{s}\sum_{k}\lambda_{kj}^{*}x_{ik}\right)}{\Psi_{ij}\left(\lambda^{*}\right)}$$

where $\lambda^{*}$ is a vector of K by (J−1) inferred Lagrange multipliers (the parameters of interest, which are equivalent to the $\beta_{kj}s$ in the previous equations), $p_{ij}^{*}$ and $w_{ijs}^{*}$ are the inferred probabilities, and $\Omega_{i}$ and $\Psi_{ij}$ are the normalization factor for the $P^{\prime }s$ and Ws respectively. If all priors are taken to be uniform, we obtain what we call the no-prior solution.

At first sight, this problem has a very large number of unknown quantities (the $p_{ij}s$ and the $w_{ijs}s$), but once we convert it into its dual representation, we see that this model is just a more flexible, generalized version of the maximum entropy and maximum likelihood approaches. It has exactly the same level of complexity: the number of λs does not change. The dual model is

(A7) $$\ell \left(\lambda \right)=\sum_{i,\;j,\;k}y_{ij}x_{ik}\lambda_{kj}-\sum_{i}\ln\left[\sum_{j}p_{ij}^{0}\exp\left(\sum_{k}x_{ik}\lambda_{kj}\right)\right]-\sum_{i,\;j}\ln\left[\sum_{s}w_{ijs}^{0}\exp\left(\sum_{k}\lambda_{kj}x_{ik}v_{s}\right)\right]\;=\sum_{i,\;j,\;k}y_{ij}x_{ik}\lambda_{kj}-\sum_{i}\ln\left[\Omega_{i}\left(\lambda \right)\right]-\sum_{i,\;j}\ln\left[\Psi_{ij}\left(\lambda \right)\right].$$

Maximizing with respect to λs yields the optimal λs, which are then inserted into Equations (A5)−(A6) above to yield the inferred probabilities, P and W. This model can be viewed as a generalized ML logit. It is computationally and statistically efficient. If the priors are uniform, and all ϵs are zero, that model is equivalent to the ML logit. However, because our constraints (moment conditions) are specified as flexible constraints, we have a much more flexible model that dominates the ML logit for all finite samples. Derivations of the covariance and all statistical tests are easily computed. See, for example, the derivations detailed in [10]. Most statistical and econometric software, such as SAS (Version 9.4), STATA (Version 17), and NLOGIT (Version 6), include the codes for that problem. For examples and specific codes, see http://info-metrics.org/index.html (accessed on 23 September 2024).
