# Supplementary material for: An Information-Theoretic Method for Identifying Effective Treatments and Policies at the Beginning of a Pandemic
Source: Entropy (Basel). 2024 Nov 26;26(12):1021. doi: 10.3390/e26121021 (PMC11727047; doi:10.3390/e26121021)
Supplement: Supplementary file 1 [file entropy-26-01021-s001.zip › Golan et al Pandemic Supplement (No Data) Revised Formatted.pdf]

Supplemental Material: An Information-Theoretic Method for Identifying Effective Treatments and Policies at the Beginning of a Pandemic

Amos Golan[1,2*], Tinatin Mumladze[1], Jeffrey M. Perloff[3], Danielle Wilson[1]

The supplementary materials include:

(1) Table S1: Data definitions and sources.

(2) S1 File: The data dictionary for cleaned data from the Open COVID Data Group [1].

(3) S2 File: Our cleaned, full dataset in both xlsx and dta form.

(4) S3 File: Our cleaned, usable subsample (485 observations) from the Open COVID Data Group [1] in both xlsx and dta form.

(5) S4 File: The main do file for the original model [2].

(6) S5 File: The necessary STATA ado file needed to run the original model. (The ado file, published by STATA, was written by Paul Corral, Senior Economist at the World Bank.)

(7) A few additional reported experiments.

Affiliations:
[1]Department of Economics, American University, Washington, D.C. 20016
[2]Santa Fe Institute, Santa Fe, NM, 87501
[3]Department of Agricultural & Resource Economics, University of California Berkeley, Berkeley CA, 94720

Address for correspondence:
Amos Golan; 4400 Massachusetts Avenue, NW, Washington, DC, United States, 20016; agolan@american.edu

Data Sources

Table S1. Data Description and Sources.

Variable	Description	Source
Original Sample from Initial Outbreak of the Pandemic to May 2020 (Sample Size of 485 Observations)		
Gender	Only kept observations whose gender (Male/Female) was known.	Open COVID-19 Data Curation Group [1]
Age	Age of observation.	
Country	Algeria, Australia, Brazil, Canada, China, Ethiopia, France, Germany, Guyana, Italy, Korea, Malaysia, Nepal, Philippines, Romania, Singapore, Switzerland, Thailand, United States and Vietnam.	
Died	Binary variable identifying observations who died from COIVD-19.	
Frequency Data from the Outbreak of the Pandemic to December 2020 (Sample of ~12.6 million COVID Cases)		
Gender	Male/Female.	COVerAGE-DB Team lead by Riffe and Acosta [3]
Country	Afghanistan, Argentina, Australia, Austria, Belgium, Canada, Cameroon, Chile, China, Colombia, Denmark, France, Germany, Greece, India, Iraq, Israel, Italy, Japan, Kenya, Korea, Maldives, Mexico, Nigeria, Norway, Panama, Peru, Philippines, Portugal, Slovenia, Spain, Ukraine, and United States	
Age	Ages categorized into intervals of five years.	
Death to Case Ratio	Produced using the estimated number of cases and deaths by the team.	
Control Variables (Country Level)		
BCG Never	Binary variable identifying observations who reside in a country that had never had a BCG vaccination program (as of 2017).	BCG World Atlas [4]
BCG Past	Binary variable identifying observations who reside in a country that previously have a BCG vaccination program but as of 2017 did not.	
BCG Universal	Binary variable identifying observations who reside in a country that as of 2017 had a universal BCG vaccination program.	
Domestic Private Health Expenditure	Health expenditure per capita in international dollars at the purchasing power parity.	World Bank [5]
Measles Immunization Rate	Percent of children between 12-23 months who received one dose of the measles vaccine before their first birthday.	
Hepatitis B Immunization Rate	Percent of children between 12-23 months who received three doses of the hepatitis B vaccine before their first birthday.	
Mortality Rate Associated with Air Pollution	Mortality rate attributed to household and ambient air pollution each year (per 100,000 in population).	

Stata Codes

Stata do file

```
clear

cd Z:\Desktop
use coviddata_clean_4May2020.dta

*Install gmentropylogit or have .ado file in the same directory

*Creating a binary outcome variable
gen died1=.
replace died1=0 if newoutcome==2
replace died1=1 if newoutcome==1

***Binary model without priors
gmentropylogit died1 nsex nage bcg_nev bcg_past immsl ihepb phexp diehh
gmentropylogit died1 nsex nage bcg_nev bcg_past immsl ihepb phexp diehh, mfx gen(pred_nopr)


***Uniform priors should produce the same results as a Binary model without priors
gen pr0=0.5
gmentropylogit died1 nsex nage bcg_nev bcg_past immsl ihepb phexp diehh, priors(pr0)
gmentropylogit died1 nsex nage bcg_nev bcg_past immsl ihepb phexp diehh, mfx priors(pr0) gen(pred_uni)

***Introducing priors by SEX and AGE of SARS W/O MEDICAL personnel
gen prsars=0.5
*Priors for MALE: nsex==0
replace prsars=0.077 if nsex==0 & nage<=44
replace prsars=0.326 if nsex==0 & nage>44 & nage<=74
replace prsars=0.647 if nsex==0 & nage>74
*Priors for FEMALE: nsex==1
replace prsars=0.037 if nsex==1 & nage<=44
replace prsars=0.245 if nsex==1 & nage>44 & nage<=74
replace prsars=0.636 if nsex==1 & nage>74

***Binary model with priors
gmentropylogit died1 nsex nage bcg_nev bcg_past immsl ihepb phexp diehh, priors(prsars)
gmentropylogit died1 nsex nage bcg_nev bcg_past immsl ihepb phexp diehh, mfx priors(prsars) gen(pred_sars)
```

Stata ado file

```
*! gmentropylogit 1.0.1 November 10, 2013 PC & MT

/*Written by Paul Corral, Senior Economist at the World Bank.
https://github.com/pcorralrodas/gmentropylogit
*/

cap prog drop gmentropylogit
program define gmentropylogit, eclass
        version 11.2
        syntax varlist(min=2 numeric fv) [if] [in] [,Mfx GENerate(string) Priors(varlist numeric max=1)]


//Mark the estimation sample

marksample touse


// Check to see if first variable is binary

        tokenize `varlist'

        qui: tab `1' if `touse'
        if r(r)!=2{
        display as error "You must specify a binary variable"
        error 498
        }

        if ("`priors'"!=""){
                assert inrange(`priors',0,1)
           replace `touse' = 0 if missing(`priors')
        }
        else{
           tempvar priors
                gen `priors' = 1/2
        }



// Local for dependent variable
local dep1 `1'
tempvar dep2
qui:gen byte `dep2'=`dep1'==0 if `touse'

local depvars `dep1' `dep2'

// obtain the independent variables
macro shift
local indeps `*'

//Remove collinear exolanatory vars
_rmcoll `indeps' if `touse', forcedrop
local indeps  `r(varlist)'
```

```
	if "`mfx'"=="mfx" {
		//Indicate dummy variables for MFX
		local words=wordcount("`indeps'")
		tempname dummy
		matrix `dummy'=J(1,`words',0)

		forvalues x= 1/`words'{
			capture assert ``x''==1 | ``x''==0
				if _rc==0 {
					qui: tab ``x''
					if r(r)==2{
						matrix `dummy'[1,`x']=1
					}
				}
		}
		mata: gme_discretemfx("`depvars'", "`indeps'", "`dummy'", "`priors'", "`touse'")
	}
	else{
		mata: gme_discrete("`depvars'", "`indeps'", "`dummy'", "`priors'", "`touse'")
	}


tempname b b2 V


mat `b' = r(beta)
mat `V' = r(V)
mat `b2'= r(beta2)

// Predicted values
if "`generate'"!=""{
	tokenize `generate'

	local wc=wordcount("`generate'")
	if `wc'!=1{
		display as error "You must specify a name for predicted variable, only one"
			}
	else{
		capture confirm name `1'
		if _rc!=0{
			display as error "Invalid name for new variable"
				}
		else{
			capture confirm variable `1'
			if _rc==0{
			display as error "For predicted values, specify variable not already in use"
				}
			else{
			qui: gen `generate'=. if `touse'

				if "`mfx'"==""{
						mata:predict_gme("`indeps'", "`b'", "`generate'", "`priors'",
"`touse'")
							}
				else{
```

```
                        mata:predict_gme("`indeps'", "`b2'", "`generate'", "`priors'", "`touse'")
                        }
                }
                }
        }
    }



// Matrix for results


mat colnames `b' = `indeps' _cons
mat colnames `V' = `indeps' _cons
mat rownames `V' = `indeps' _cons

// Number of observations
local N = r(N)

ereturn post `b' `V', depname(`dep1') obs(`N') esample(`touse')

// Statistics

//Number of observations
ereturn scalar N = r(N)
//Degs of freedom
ereturn scalar d_fm = (r(K)-1)
//Log likelihood
ereturn scalar lnf = r(lnf)
//Log likelihood
ereturn scalar lnf0 = r(lnf0)
//Normalized entropy
ereturn scalar Sp = r(Sp)
//Pseudo R2
ereturn scalar R2 = (1- r(Sp))
//Entropy for probs.
ereturn scalar S = r(S)
//Entropy ratio statistic
ereturn scalar ERS = 2*r(N)*ln(2)*(1- r(Sp))
// P value for LR
ereturn scalar pv = chiprob(e(d_fm),e(ERS))

//        Generate correct prediction percent

if "`generate'"!=""        {
tempvar correct predicted
qui: gen byte `predicted' = `generate'>=0.5
qui: gen byte `correct' = (`predicted'==1 & `dep1'==1) | (`predicted'==0 & `dep1'==0)
qui: sum `correct'
ereturn scalar pred = r(mean)*100
}


/// Result table
```

```
if "`mfx'"!=""{
display _newline in gr "Generalized Maximum Entropy (Logit), dF/dx" _col(52) in gr "Number of obs" _col(71) in gr "=" _col(72) in ye %7.0f e(N)
}
else{
display _newline in gr "Generalized Maximum Entropy (Logit)" _col(52) in gr "Number of obs" _col(71) in gr "=" _col(72) in ye %7.0f e(N)
}

display _col(52) in gr "Degrees of freedom" _col(71) in gr "=" _col(72) in ye %7.0f e(d_fm)
display _col(52) in gr "Entropy for probs." _col(71) in gr "=" _col(72) in ye %7.1f e(S)
display _col(52) in gr "Normalized entropy" _col(71) in gr "="  _col(72) in ye %7.4f e(Sp)
display _col(52) in gr "Ent. ratio stat." _col(71) in gr "="  _col(72) in ye %7.1f e(ERS)
display _col(52) in gr "P Val for LR" _col(71) in gr "="  _col(72) in ye %7.4f e(pv)
display _col(1) in gr "Criterion F (log L) = "  in ye e(lnf) _col(52) in gr "Pseudo R2" _col(71) in gr "=" _col(72)  in ye %7.4f e(R2)
ereturn display
if "`mfx'"!=""{
display _col(1) in gr "Partial effect for dummy is E[y|x,d=1] - E[y|x,d=0]"
}
if "`generate'"!=""{
display _col(1) in gr "Percent correctly predicted:"  in ye e(pred)
}


end

*mata:mata clear
version 11.2
mata: mata set matastrict on
mata:
// Discrete GME 1.0.0  Nov. 24, 2013
void predict_gme (string scalar xname,
		string scalar bname,
						string scalar pname,
						string scalar prior,
		string scalar touse)

{

	real matrix X
	real matrix Po
	real vector  beta, newvar

	X =st_data(., tokens(xname), touse)
	Po=st_data(., tokens(prior), touse)
	Po = Po,(1:-Po)
	X=X,J(rows(X),1,1)
	st_view(newvar,., tokens(pname), touse)
	beta=st_matrix(bname)

	newvar[.,1]=(Po[.,1]:*exp(quadcross(X',beta'))):/((Po[.,2]+Po[.,1]:*exp(quadcross(X',beta'))))
}
end
*mata:mata clear
version 11.2
```

```
mata: mata set mataoptimize on
mata: mata set matastrict off

mata:

//Discrete GME optimization 1.0.0 Nov. 24, 2013

function MEdiscrete(todo, R, Y, X, v, Po, L, g, H)
{
        PSI=J(rows(Y), cols(Y),rows(v))
        B=J(1,cols(X),0)\colshape(R, cols(X))

        P1=quadcross(X',B')

        w0 = 1/3,1/3,1/3
        for (i=2; i<=cols(P1); i++){
                PSI[.,i]=quadrowsum(w0:*exp(-(quadcross(P1[.,i]',v'))))
        }

        P=quadrowsum((Po:*exp(-P1)))

        L=-(quadsum(quadcolsum((P1):*Y))+quadsum(ln(P))+quadsum(ln(PSI)))
}
end
*mata:mata clear
version 11.2
mata: mata set matastrict on
mata:
// Discrete GME 1.0.1  Dec. 15, 2013, Author: Paul Corral
void gme_discretemfx(string scalar yname,
                                        string scalar xname,
                                        string scalar dname,
                                        string scalar priors,
                                        string scalar touse)

{

        real matrix  Y, X, vcov, dfdz, dfdB, G, dummy, x1, x0, vcov2, Po1
        real vector beta, v1, p, PxP, MFX, p1, p0, f1, f0, grad
        real scalar K, N, s, lnf, lnf0, i, S, Sp

        // Use st_data to import variables from stata
        Y=st_data(., tokens(yname), touse)
        X=st_data(., tokens(xname), touse)
        Po1=st_data(., tokens(priors), touse)
        Po1 = Po1,(1:-Po1)
        //Add constant term
        X = X, J(rows(X),1,1)

        // Observations
        N=rows(X)
        // Variables
        K=cols(X)

        // Import matrix dummy from stata
        dummy=st_matrix(dname)
```

```
// Create error  vector, Symmetric error support vector

v1=-1/sqrt(N)\0\1/sqrt(N)

// Optimization
s=optimize_init()
optimize_init_evaluator(s,&MEdiscrete())
optimize_init_evaluatortype(s,"d0")
optimize_init_which(s,"max")
optimize_init_singularHmethod(s, "hybrid")
optimize_init_argument(s,1,Y)
optimize_init_argument(s,2,X)
optimize_init_argument(s,3,v1)
optimize_init_argument(s,4,Po1)
optimize_init_valueid(s, "log likelihood")

optimize_init_params(s,J(1,cols(X),0))
beta=optimize(s)
vcov=optimize_result_V_oim(s)
lnf=optimize_result_value(s)
lnf0=optimize_result_value0(s)

// MFX: Generate Probabilities
p=(Po1[.,1]:*exp(quadcross(-X',beta'))):/(Po1[.,2]:+Po1[.,1]:*exp(quadcross(-X',beta')))
PxP = p:*(J(rows(p),cols(p),1)-p)
MFX = mean(quadcross(PxP',beta))

// Delta method for MFX covar
dfdz = (J(N,1,1)-p:*2):*PxP
dfdB=X:*dfdz
G = quadcross(beta,mean(dfdB))

for(i=1; i<=K; i++)          G[i,i]=G[i,i]+mean(PxP)

//For Dummies
for(i=1; i<=cols(dummy); i++){
        if (dummy[,i]==1) {
                x1 = X
                x1[,i] = J(N,1,1)
                x0 = X
                x0[,i] = J(N,1,0)
                beta


                p1 =
(Po1[.,1]:*exp(quadcross(x1',beta'))):/(Po1[.,2]+(Po1[.,1]:*exp(quadcross(x1',beta'))))

                p0 =
(Po1[.,1]:*exp(quadcross(x0',beta'))):/(Po1[.,2]+(Po1[.,1]:*exp(quadcross(x0',beta'))))

                MFX[,i] = mean(p1) - mean(p0)

                f1 = mean(x1:*((-p1:+1):*p1))
                f0 = mean(x0:*((-p0:+1):*p0))
```

```
                        grad = f1 - f0
                        grad[,i] = f1[,i]

                        G[i,] = grad
                }
        }

                // Covariance matrix for MFX
                vcov2 = G*vcov*G'

        // Normalized entropy
        S = -(sum(p:*log(p))+sum((1:-p):*log(1:-p)))
        Sp = S/(rows(X)*log(cols(Y)))

        // Return results to stata
        st_matrix("r(beta)", MFX)
        st_matrix("r(V)", vcov2)
        st_matrix("r(beta2)", beta)
        st_numscalar("r(lnf)", lnf)
        st_numscalar("r(lnf0)", lnf0)
        st_numscalar("r(N)", N)
        st_numscalar("r(K)", K)
        st_numscalar("r(Sp)", Sp)
        st_numscalar("r(S)", S)

}

end

*mata:mata clear
version 11.2
mata: mata set matastrict on
mata:
// Discrete GME 1.0.0  Nov. 24, 2013
void gme_discrete(string scalar yname,
            string scalar xname,
            string scalar dummy,
                                        string scalar priors,
            string scalar touse)

{

        real matrix  Y, X, vcov, Po1
        real vector cons, beta, v1, P
        real scalar K, N, lnf, lnf0, s, Sp, S

        // Use st_data to import variables from stata

        Y  =st_data(., tokens(yname), touse)
        X  =st_data(., tokens(xname), touse)
        Po1=st_data(., tokens(priors), touse)
        Po1 = Po1,(1:-Po1)
        //Add constant term
        X = X,J(rows(X),1,1)

        // Observations
```

```
        N=rows(X)
        // Variables
        K=cols(X)

        // Create error  vector, Symmetric error support vector
        v1=-1/sqrt(rows(Y))\0\1/sqrt(rows(Y))

        // Optimization
        s=optimize_init()
        optimize_init_evaluator(s,&MEdiscrete())
        optimize_init_evaluatortype(s,"d0")
        optimize_init_which(s,"max")
        optimize_init_singularHmethod(s, "hybrid")
        optimize_init_argument(s,1,Y)
        optimize_init_argument(s,2,X)
        optimize_init_argument(s,3,v1)
        optimize_init_argument(s,4,Po1)
        optimize_init_valueid(s, "log likelihood")

        optimize_init_params(s,J(1,cols(X),0))
        beta=optimize(s)
        vcov=optimize_result_V_oim(s)
        lnf=optimize_result_value(s)
        lnf0=optimize_result_value0(s)

        // Normalized entropy

        P=(Po1[.,1]:*exp(quadcross(-X',beta'))):/(Po1[.,2]:+Po1[.,1]:*exp(quadcross(-X',beta')))

        S=-(sum(P:*log(P))+sum((1:-P):*log((1:-P))))
        Sp=S/(rows(X)*log(cols(Y)))

        // Return results to stata
        st_matrix("r(beta)", beta)
        st_matrix("r(V)", vcov)
        st_numscalar("r(lnf)", lnf)
        st_numscalar("r(lnf0)", lnf0)
        st_numscalar("r(N)", N)
        st_numscalar("r(K)", K)
        st_numscalar("r(Sp)", Sp)
        st_numscalar("r(S)", S)
}
end
```

Additional Reported Experiments

Pittet et al. [6] reported a randomized BCG trial to protect against COVID-19 in healthcare workers in Australia, the United Kingdom, the Netherlands, Spain, and Brazil. They did not find a statistically significant effect. The subgroup who received the vaccination in the past had a lower risk for a severe COVID-19 reaction. However, three-quarters of individuals in the trial lived in countries with a current or former mandatory BCG policy. The study did not control for that impact. Moreover, these workers probably had better healthcare than others, three-quarters were female (who are less at risk), and few in either the control or experimental group were hospitalized. Most importantly, only one participant out of nearly 4,000 died.

They speculated that the BCG may also provide protection against other infectious diseases and its protection may last decades. These studies show that the BCG vaccine may provide good protection against COVID-19 and its emerging variants. Though there is more to learn about the effect of BCG vaccine on protecting COVID-infected individuals (e.g., Doesschate et al. [7]), it seems that the results of a well-designed and proctored experiments are consistent with very early results with extremely sparse data that was analyzed using the information-theoretic approach we discussed here. See also a summary of recent findings in an August 16, 2022 article in the New York Times [8].

References

1. Open COVID-19 Data Working Group. Detailed Epidemiological Data from the COVID-19 Outbreak. 2020. https://github.com/beoutbreakprepared/nCoV2019
2. Golan A.; Mumladze T.; Wilson D.; et al. Effect of Universal TB Vaccination and Other Policy-Relevant Factors on the Probability of Patient Death from COVID-19, 2020. https://hceconomics.uchicago.edu/research/working-paper/effect-universal-tb-vaccination-and-other-policy-relevant-factors
3. Riffe T.; Acosta E.; Acosta E.J.; et al. Data Resource Profile: COVerAGE-DB: a global demographic database of COVID-19 cases and deaths. *Int J Epidemiol* **2021**, 50, 390–390, https://doi.org/10.1093/ije/dyab027.
4. Zwerling A.; Behr M.A.; Verma A.; et al. The BCG World Atlas: A Database of Global BCG Vaccination Policies and Practices. *PLoS Med* **2011**, 8, 1-8, https://doi.org/10.1371/journal.pmed.1001012.
5. World Bank Open Data.
6. Pittet L.F.; Messina N.L.; Orsini F.; et al. Randomized Trial of BCG Vaccine to Protect against Covid-19 in Health Care Workers. *NEJM* **2023**, 388, 1582–96, https://doi.org/10.1056/NEJMoa2212616.
7. ten Doesschate T.; van der Vaart T.W.; Debisarun P.A.; et al. Bacillus Calmette-Guérin vaccine to reduce healthcare worker absenteeism in COVID-19 pandemic, a randomized controlled trial. *CMI* **2022**, 28, 1278–85, https://doi.org/10.1016/j.cmi.2022.04.009.
8. Rabin RC. Why a Century-Old Vaccine Offers New Hope Against Pathogens. New York Times. 2022. https://www.nytimes.com/2022/08/16/health/bcg-vaccine-diabetes-covid.html.
